# Inhibition of histone deacetylase 6 destabilizes ERK phosphorylation and suppresses cancer proliferation via modulation of the tubulin acetylation-GRP78 interaction

**DOI:** 10.1186/s12929-023-00898-3

**Published:** 2023-01-13

**Authors:** Onsurang Wattanathamsan, Naphat Chantaravisoot, Piriya Wongkongkathep, Sakkarin Kungsukool, Paninee Chetprayoon, Pithi Chanvorachote, Chanida Vinayanuwattikun, Varisa Pongrakhananon

**Affiliations:** 1 Department of Pharmacology and Physiology, Faculty of Pharmaceutical Sciences,, Chulalongkorn University, Bangkok, Thailand; 2 Department of Biochemistry, Faculty of Medicine, Chulalongkorn University, Bangkok, Thailand; 3 Center of Excellence in Systems Biology, Faculty of Medicine, Chulalongkorn University, Bangkok, Thailand; 4 Department of Respiratory Medicine, Central Chest Institute of Thailand, Muang District, Nonthaburi, Thailand; 5 Toxicology and Bio Evaluation Service Center, National Science and Technology Development Agency, Pathum Thani, Thailand; 6 Division of Medical Oncology, Department of Medicine, Faculty of Medicine, Chulalongkorn University, Bangkok, Thailand; 7 Preclinical Toxicity and Efficacy Assessment of Medicines and Chemicals Research Cluster, Chulalongkorn University, Bangkok, Thailand

**Keywords:** Extracellular signal-regulated kinase (ERK), Glucose-regulated protein 78 (GRP78), Histone deacetylase 6 (HDAC6), Lung cancer, Tubulin acetylation

## Abstract

**Background:**

The leading cause of cancer-related mortality worldwide is lung cancer, and its clinical outcome and prognosis are still unsatisfactory. The understanding of potential molecular targets is necessary for clinical implications in precision diagnostic and/or therapeutic purposes. Histone deacetylase 6 (HDAC6), a major deacetylase enzyme, is a promising target for cancer therapy; however, the molecular mechanism regulating cancer pathogenesis is largely unknown.

**Methods:**

The clinical relevance of HDAC6 expression levels and their correlation with the overall survival rate were analyzed based on the TCGA and GEO databases. HDAC6 expression in clinical samples obtained from lung cancer tissues and patient-derived primary lung cancer cells was evaluated using qRT–PCR and Western blot analysis. The potential regulatory mechanism of HDAC6 was identified by proteomic analysis and validated by immunoblotting, immunofluorescence, microtubule sedimentation, and immunoprecipitation-mass spectrometry (IP-MS) assays using a specific inhibitor of HDAC6, trichostatin A (TSA) and RNA interference to HDAC6 (siHDAC6). Lung cancer cell growth was assessed by an in vitro 2-dimensional (2D) cell proliferation assay and 3D tumor spheroid formation using patient-derived lung cancer cells.

**Results:**

HDAC6 was upregulated in lung cancer specimens and significantly correlated with poor prognosis. Inhibition of HDAC6 by TSA and siHDAC6 caused downregulation of phosphorylated extracellular signal-regulated kinase (p-ERK), which was dependent on the tubulin acetylation status. Tubulin acetylation induced by TSA and siHDAC6 mediated the dissociation of p-ERK on microtubules, causing p-ERK destabilization. The proteomic analysis demonstrated that the molecular chaperone glucose-regulated protein 78 (GRP78) was an important scaffolder required for p-ERK localization on microtubules, and this phenomenon was significantly inhibited by either TSA, siHDAC6, or siGRP78. In addition, suppression of HDAC6 strongly attenuated an in vitro 2D lung cancer cell growth and an in vitro 3D patient derived-lung cancer spheroid growth.

**Conclusions:**

HDAC6 inhibition led to upregulate tubulin acetylation, causing GRP78-p-ERK dissociation from microtubules. As a result, p-ERK levels were decreased, and lung cancer cell growth was subsequently suppressed. This study reveals the intriguing role and molecular mechanism of HDAC6 as a tumor promoter, and its inhibition represents a promising approach for anticancer therapy.

**Supplementary Information:**

The online version contains supplementary material available at 10.1186/s12929-023-00898-3.

## Introduction

Currently, lung cancer is the leading cause of cancer-related deaths, with a million new cases being diagnosed each year [1]. Lung cancer morbidity has continued to expand worldwide, specifically in developing countries, which is partially due to air pollution [2]. Lung adenocarcinoma is the most frequent histological subtype of pulmonary cancer [3]. The adenocarcinoma subtype is found in approximately 40% of lung cancer patients and has distinct morphological and molecular profiles arising from various lung locations [4]. Moreover, the 5-year overall survival rate of lung adenocarcinoma patients is still remarkably low [5]. Even though multiple therapeutic approaches are currently more advanced, the outcome and patient prognosis remain unsatisfactory. Therefore, the identification of potential molecular targets and their mechanisms is indispensable for exploring more precise diagnoses and therapies.

Histone deacetylases (HDACs) are important enzymes that are primarily known to regulate the acetylation status of histones [6]. Among HDACs, HDAC6 has recently attracted interest owing to its functional role in cancers [7, 8]. HDAC6 promoted lung cancer proliferation, metastasis, and chemotherapy resistance. Inhibition of HDAC6 by small molecule was able to induce apoptosis and cell cycle arrest via Notch1 signaling [9], and RNA interference to HDAC6 attenuated TGF-β-mediated epithelial to mesenchymal transition (EMT) and metastasis [10]. In addition, HDAC6 cooperating with USP10 enhanced cisplatin resistance [11]. Apart from histones, α-tubulin was reported to be a major cytoplasmic substrate of HDAC6 that has a significant impact on tumorigenesis [12, 13]. The high expression of HDAC6 was identified in breast, renal, lung, and endometrial cancers, and its upregulation in cancers was strongly associated with a poor overall survival rate [9, 14–16]. HDAC6 is required for removing the acetyl group from α-tubulin at the Lys 40 position, which is located in stabilized microtubules [12]. Evidently, HDAC6 inhibition suppressed cancer cell migration as a result of an increase in acetylated α-tubulin [17]. In addition, HDAC6 is a unique enzyme that participates in several cancer signaling pathways, including extracellular signal-regulated kinase (ERK) signaling [18–20]. The ERK pathway plays a crucial role in supporting cancer survival, growth, and metastasis [21, 22]. Previous studies showed an association between ERK signaling and HDAC6 activity. Inhibition of HDAC6 was shown to decrease the level of ERK phosphorylation [23–25]. HDAC6 is required for stabilizing the epidermal growth factor receptor (EGFR) and its activity, whose ERK is the downstream signaling [26]. In addition, HDAC6 caused microtubule deacetylation and delayed the EGFR endocytic trafficking along microtubules to the lysosome for degradation [27, 28], suggesting an association of microtubule acetylation participating in the mechanism of HDAC6-regulating signaling, however, the exact molecular mechanism regarding the interplay between deacetylase activity of HDAC6 on microtubules and ERK phosphorylation is remaining unknown. In the present study, we, therefore, investigated HDAC6 expression in human lung adenocarcinoma tissues and the underlying mechanism of ERK phosphorylation using the HDAC6 inhibitor trichostatin A (TSA) and small interference to HDAC6 (siHDAC6). We found that tubulin acetylation cooperated under this regulation, in which glucose-regulated protein 78 (GRP78) was a crucial link between tubulin and p-ERK. Tubulin acetylation induced by either TSA or siHDAC6 mediated p-ERK/GRP78 detachment from microtubules and thus destabilized p-ERK. As a result, HDAC6 inhibition contributed to attenuating in vitro 2D and 3D cancer cell growth. Our study provides an intriguing mechanism of HDAC6 inhibition, suggesting that HDAC6 is an attractive therapeutic target for lung adenocarcinoma.

## Methods

### Bioinformatic evaluation of HDAC6 gene expression

The expression of HDAC6 in lung adenocarcinoma and normal adjacent tissues was obtained from the Genome Atlas project (TCGA) in the cBioPortal database (http://cbioportal.org) and Gene Expression Omnibus (http://www.ncbi.nlm.nih.gov/geo/) in the GEO database (GSE27262). HDAC6 mRNA expression and survival correlation data of lung adenocarcinoma were downloaded from the GEO database (GSE50081). Groups according to the median expression of HDAC6 (high vs. low expression) were analyzed for overall survival (OS) and presented as Kaplan–Meier plots by Prism 8 (GraphPad).

### Clinical samples

All 43 samples of primary malignant lung tissues and benign lung tissues were obtained during surgical resection at the Central Chest Institute of Thailand, Muang District, Nonthaburi, Thailand. Written informed consent was obtained from all participants before the start of the study. All studies were approved by the Ethical Committee of the Central Chest Institute of Thailand (086/2563) and performed in accordance with relevant regulations and guidelines.

### Cell culture

Non-small cell lung cancer H460 and A549 cells and normal lung epithelial BEAS-2B cells were obtained from the American Type Culture Collection (ATCC; Manassas, VA, USA). Patient-derived lung cancer cells (ELC12, ELC16, ELC17, and ELC20) were isolated from the pleural effusions of recurrent or advanced-stage non-small cell lung cancer patients who had been diagnosed at King Chulalongkorn Memorial Hospital [29]. The protocol of conduction was approved by the Ethics Committee of the Faculty of Medicine, Chulalongkorn University, Bangkok, Thailand (IRB 365/62), and informed consent was obtained from all participants. This study was carried out in accordance with the principles of the World Medical Association Declaration of Helsinki. All cells were maintained in RPMI or DMEM supplemented with 10% fetal bovine serum, 1% l-glutamine, and 1% penicillin/streptomycin at 37 °C and 5% CO_2_.

### Protein–protein network construction

The cancer signaling-related protein network of HDAC6 and ERK was constructed by the Search Tool for the Retrieval of Interacting Genes/Proteins (STRING; https://string-db.org/) with a 0.4 medium confidence score. The protein–protein interaction network of significantly associated proteins was constructed and presented with the combined score [30].

### RNA interference (RNAi) and transfection

The knockdown experiment was performed using Lipofectamine® RNAiMAX following the manufacturer’s instructions (Invitrogen, MA, USA). Stealth RNAi and the control were purchased from Invitrogen (Invitrogen, MA, USA). The sequence of siHDAC6 was 5′-GGATGGATCTGAACCTTGAGA-3′, and the sequence of siGRP78 was 5′-AAGGTTACCCATGCAGTTGTT-3′. Briefly, 25, and 50 nM of siRNA in OptiMEM (Invitrogen, MA, USA) were incubated with Lipofectamine® RNAiMAX in OptiMEM at room temperature for 15 min. The mixture was added in a drop-wise fashion onto the cells and incubated further at 37 °C for 6 h. After transfection for 72 h, transfection efficiency was assessed by qRT–PCR or Western blot analysis, prior to other biochemical assays.

### RNA extraction and quantitative real-time polymerase chain reaction (qRT–PCR)

RNA was extracted using GENEzol reagent (Geneaid Biotech, Taipei, Taiwan). One microgram of RNA was reverse transcribed to cDNA using SuperScript™ III Reverse Transcriptase (Invitrogen, MA, USA). The mRNA expression levels of target genes were determined using a Step one plus real-time PCR system and the SensiFAST™ SYBR® NO-ROX Kit (Bioline, London, UK). The primer sequences are listed in Additional file 1: Table S1. Real-time PCR was performed using a StepOnePlus Real-Time PCR system (Applied Biosystems, CA, USA). The thermocycling conditions were set as follows: 95 °C for 10 min, 95 °C for 30 s (40 cycles), and 60 °C for 30 s. The data were analyzed using the Ct^−ΔΔ^ method [31].

### Western blot analysis

Cells were lysed with TMN buffer (20 mM Tris–HCl, pH 7.5; 1 mM MgCl_2_; 150 mM NaCl; 20 mM NaF; 0.5% sodium deoxycholate; 1% nonidet-40; 0.1 mM phenylmethylsulfonyl fluoride; and cOmpleteTM protease inhibitor cocktail (Roche, Basel, Switzerland)) on ice for 30 min. The supernatant was collected by centrifugation at 12,000×*g* at 4 °C for 20 min. Protein content was measured by a BCA Protein Assay Kit (Pierce™, Thermo Fisher Scientific, CA, USA). Equal amounts of protein were dissolved in SDS polyacrylamide gel electrophoresis (SDS-PAGE) and transferred to PVDF membranes. Blots were incubated with 5% skim milk in TBS-T buffer (Tris buffer saline with 0.075% Tween-20) at room temperature for 1 h, specific primary antibody at 4 °C overnight, and corresponding secondary antibody at room temperature for 2 h. An equal loading was confirmed by GAPDH or tubulin. Protein signals were detected by a chemiluminescence system (Merck Millipore, MA, USA). The relative protein intensity was analyzed and normalized to the loading control by ImageJ software (NIH). The antibodies used are provided in Additional file 1: Table S2.

### Immunofluorescence assay

Cells were fixed with cold methanol at − 20 °C for 5 min. After that, cells were permeabilized in 0.1% Triton X-100 in PBS, blocked with 3% BSA, and incubated with primary antibody at 4 °C overnight and secondary antibody at room temperature for 2 h in the dark. Images were observed using a confocal microscope (Zeiss LSM 900, Jena, Germany) with a 20× or 63× oil immersion objective lens. The colocalization of fluorescence signals obtained from at least 20 cells was analyzed using ImageJ software (NIH) with the JACoP plugin.

### Microtubule sedimentation assay

Cells were incubated with 1 μM Taxol at 37 °C for 30 min and dissolved in a microtubule-stabilizing buffer containing 80 mM PIPES, 80 mM K-1,4-piperazinediethanesulfonic acid (pH 6.8), 1 mM EGTA, 1 mM MgCl_2_, 0.5% (vol/vol) nonidet P-40, 20 mM NaF, 0.5% sodium deoxycholate, 10 mM Taxol, 0.1 mM phenylmethylsulfonyl fluoride, and cOmpleteTM protease inhibitor cocktail (Roche, Basel, Switzerland) at 37 °C for 5 min in the dark. The microtubule fraction was separated by centrifugation at 18,000×*g* at 30 °C for 15 min. The pellet was washed with microtubule-stabilizing buffer without detergent and resuspended with the same buffer in an equal volume of supernatant. Both pellet and supernatant fractions were boiled in a sampling buffer at 95 °C for 10 min and analyzed by Western blot analysis.

### Immunoprecipitation assay

Cells were dissolved in lysis buffer, and the supernatant was collected by centrifugation at 20,000×*g* and 4 °C for 20 min. Then, a pre-clearing step was performed using Protein G-conjugated Sepharose beads (GE Healthcare, IL, USA) at 4 °C for 1 h. The supernatant was then separated by centrifugation and incubated with a specific antibody or IgG as the control at 4 °C overnight. The protein complexes were pulled down by incubation with Protein G-conjugated Sepharose beads at 4 °C for 1 h. After washing, the precipitate was heated in a sample buffer at 95 °C for 5 min and subjected to Western blot analysis.

### Immunoprecipitation and mass spectrometry assay (IP-MS)

Coimmunoprecipitation was performed by pulling down ERK using an anti-ERK antibody. The ERK preparations were fractionated by SDS–PAGE, and gels were stained using Coomassie blue to detect and excise specific bands for mass spectrometry analysis. Proteins were reduced with 10 mM dithiothreitol and alkylated with 50 mM iodoacetamide. In-gel trypsin digestion was performed at 37 °C, and proteins were identified by liquid chromatography-tandem mass spectrometry (LC–MS/MS) as previously described [32]. The data were analyzed using Proteome Discoverer 2.1 (Thermo Fisher Scientific, CA, USA) and searched against a UniProt human protein database (UP000005640, 20370 entries) to identify proteins entrapped in gel slices. At least three unique peptides were identified, and pathway analysis was evaluated using the Reactome pathway database [33].

### Cell proliferation assay

A total of 5 × 10^3^ cells/well were seeded onto 96-well plates for 24, 48, and 72 h. Cells were incubated with MTT solution (0.5 mg/mL) at 37 °C for 4 h. The absorbance at 570 nm was measured using a microplate reader (Perkin Elmer VICTOR3/Wallac 1420). The proliferation was calculated relative to the initial time of each group.

### Three-dimensional (3D) tumor spheroid formation assay

Cells were seeded at a density of 7 × 10^3^ per well into 96-well round-bottom ultralow attachment plates (Corning, NY, USA). Spheroids were imaged every day for up to 5 d by a Meiji Techno TC5100 inverted microscope (Saitama, Japan) with a Tuscan camera and TCapture software (version 4.3.0.605). The diameter of spheroids was measured by ImageJ software (NIH). Spheroid growth was calculated as a relative tumor spheroid growth [34].

### Statistical analysis

All the data are presented as the means ± SEM obtained from at least three independent experiments. Statistical analysis between groups was performed using an unpaired Student’s *t-test* or the Mann–Whitney *U test* using Prism 8 (GraphPad software). One-way ANOVA with Tukey’s multiple comparison test was applied to determine the statistical significance among groups. p-values less than 0.05 were considered statistically significant.

## Results

### HDAC6 was overexpressed in lung adenocarcinoma

**Fig. 1:**
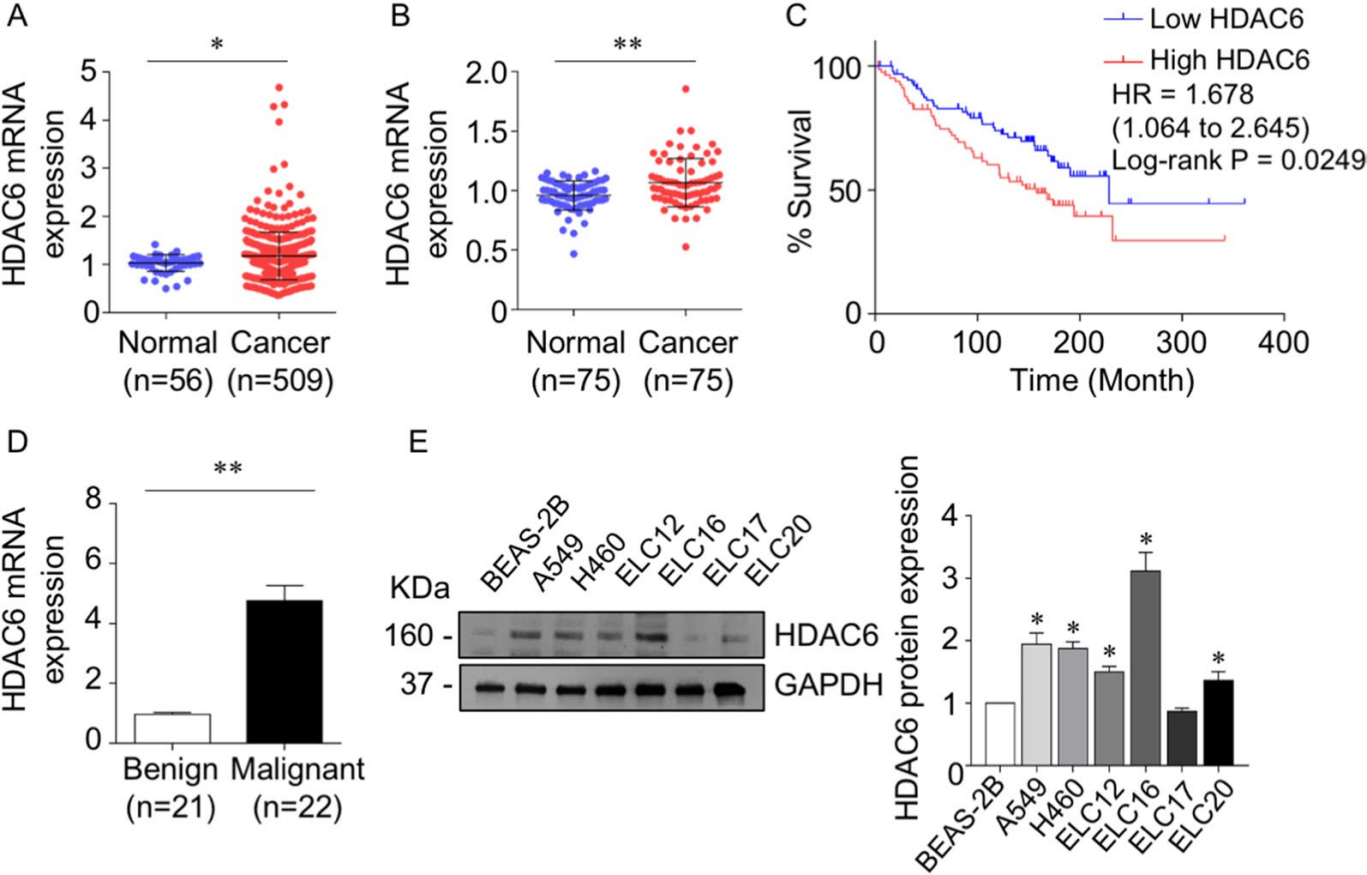
HDAC6 was overexpressed in lung adenocarcinoma. **A** HDAC6 mRNA levels show a high abundance in lung adenocarcinoma tissues compared to normal lung adjacent tissues based on the TCGA database and **B** GEO database (GSE27262). **C** Kaplan–Meier survival analysis according to HDAC6 levels in a lung adenocarcinoma dataset obtained from GEO (GSE50081). **D** Relative mRNA expression levels of HDAC6 in clinical lung malignant and benign tissues were quantified by qRT–PCR. Data are presented as the mean ± SEM. *p < 0.05; **p < 0.01. **E** Protein expression levels of HDAC6 in non-small cell lung cancer cells (H460 and A549), patient-derived malignant cancer cells (ELC12, 16, 17, and 20), and normal lung epithelial cells (BEAS-2B) were quantified by Western blot assay. The intensity was normalized to that of GAPDH. Data are presented as the mean ± SEM. *p < 0.05 vs. BEAS-2B cells (*n* = *3*)

To determine the HDAC6 expression level in lung adenocarcinoma, we queried the TCGA lung adenocarcinoma database for the variant expression of HDAC6 between tumor and normal lung adjacent tissues. The data revealed that the mRNA levels of HDAC6 were substantially increased in lung adenocarcinoma tissues (n = 509) compared with normal lung tissues (n = 56) (Fig. 1A). Moreover, the average expression level of the HDAC6 gene obtained from the GEO database (GSE27262) was significantly higher in lung cancer tissues than in adjacent nontumorous lung tissues (Fig. 1B). Next, we investigated whether HDAC6 was related to prognosis in lung adenocarcinoma. The Kaplan–Meier plot demonstrated that patients with high HDAC6 expression had a poorer prognosis than those with low HDAC6 expression (hazard ratio (HR) = 1.678, p value = 0.0249) (Fig. 1C). The clinical expression of HDAC6 in lung malignancy was further explored. The results demonstrated that HDAC6 mRNA expression levels were dramatically increased in malignant tissues (Fig. 1D), whereas there were no significant correlations of clinicopathological parameters (Additional file 1: Table S3). Consistent with patient-derived primary lung cancer cells (ELC12, ELC16, ELC17, and ELC20) and lung cancer cell lines (H460 and A549), most of them exhibited an upregulation of HDAC6 protein expression compared with normal lung epithelial BEAS-2B cells (Fig. 1E), suggesting that HDAC6 is a potential therapeutic target in lung adenocarcinoma.

### HDAC6 inhibition by trichostatin A (TSA) and RNA interference attenuated ERK phosphorylation through tubulin acetylation

**Fig. 2:**
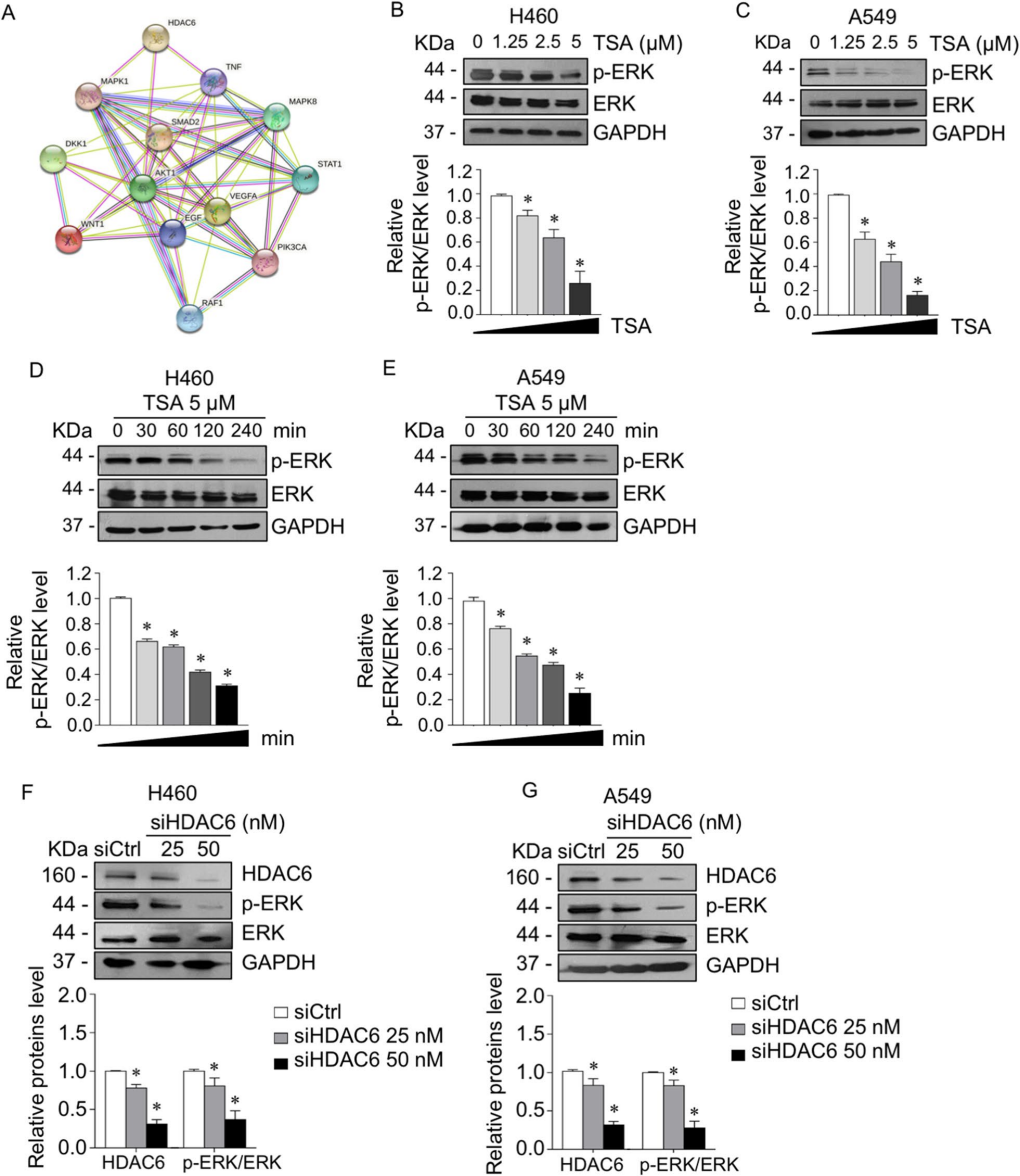
Suppression of HDAC6 downregulated ERK phosphorylation. **A** The protein–protein interaction network of HDAC6 and cancer-related proteins was constructed using the STRING online database. **B** H460 and **C** A549 cells were treated with various concentrations of TSA for 4 h. **D** H460 and **E** A549 cells were treated with 5 µM TSA for various time points. p-ERK and ERK expressions were examined by Western blot assay. The intensity was normalized to that of GAPDH. *p < 0.05 vs. control cells (*n* = *3*). **F** H460 and **G** A549 cells were transfected with siRNA against HDAC6 (siHDAC6) or control siRNA (siCtrl). The protein expressions of HDAC6, p-ERK, and ERK were analyzed by immunoblotting. The intensity was normalized to that of GAPDH. Data are presented as the mean ± SEM. *p < 0.05 vs. siCtrl cells (*n* = *3*)

To identify the target signaling of HDAC6, a protein–protein interaction (PPI) network of HDAC6 and cancer-related signaling was constructed based on information from the STRING database. Figure 2A and Additional file 1: Table S4 show the construction of a graphical network and interpretable combined score, indicating whether a proposed connection is biologically meaningful given all the contributing evidence [35]. Based on the combined score of each interaction with 0.4 confidence, the top 3 significant cancer-related signaling pathways in association with HDAC6 were Akt1 (0.704), MAPK1 (0.584), and TNF (0.403). Since the relevance of HDAC6 and Akt1 has been comprehensively investigated [36–38] and its regulation of ERK (MAPK1) was not fully explored, the involvement of HDAC6 and ERK was investigated. H460 and A549 lung adenocarcinoma cells were treated with trichostatin A (TSA), an HDAC6 inhibitor, or transfected with siHDAC6. Western blot analysis revealed that p-ERK was gradually suppressed in a dose- (Fig. 2B, C) and time-dependent manner (Fig. 2D, E) in response to TSA. Consistency, HDAC6 knockdown downregulated ERK phosphorylation (Fig. 2F, G). These results confirmed that ERK signaling is an intriguing downstream target of HDAC6 in lung adenocarcinoma cells.

**Fig. 3:**
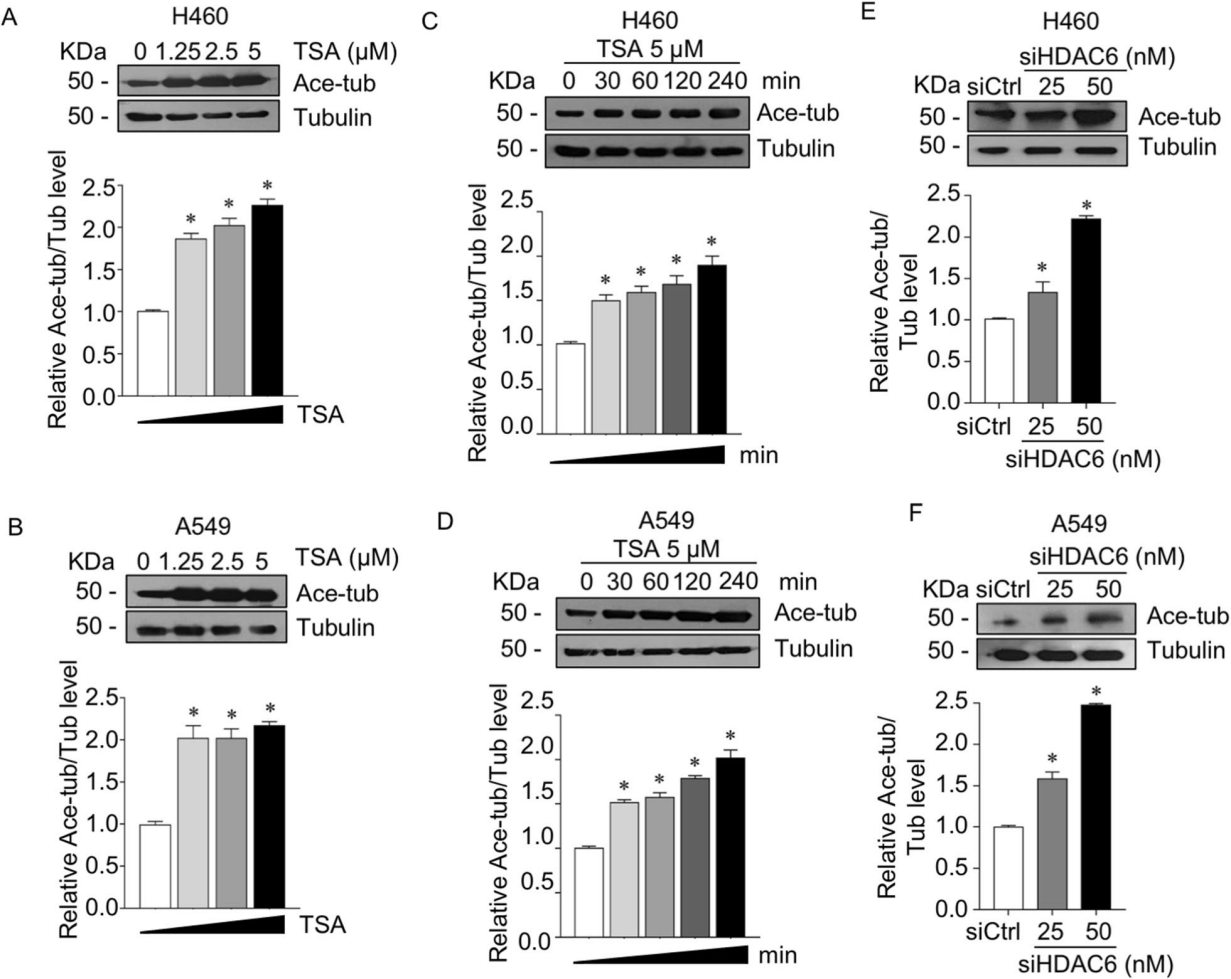
Suppression of HDAC6 upregulated tubulin acetylation. **A** H460 and **B** A549 cells were treated with various concentrations of TSA for 4 h. **C** H460 and **D** A549 cells were treated with 5 µM TSA for various time points. Acetylated tubulin expression was examined by immunoblotting. The intensity was normalized to that of α-tubulin. *p < 0.05 vs. control cells (*n* = *3*). **E** H460 and **F** A549 cells were transfected with siRNA against HDAC6 (siHDAC6) or control siRNA (siCtrl). Acetylated tubulin expression was analyzed by immunoblotting. The intensity was normalized to that of α-tubulin. Data are presented as the mean ± SEM. *p < 0.05 vs. siCtrl cells (*n* = *3*)

**Fig. 4:**
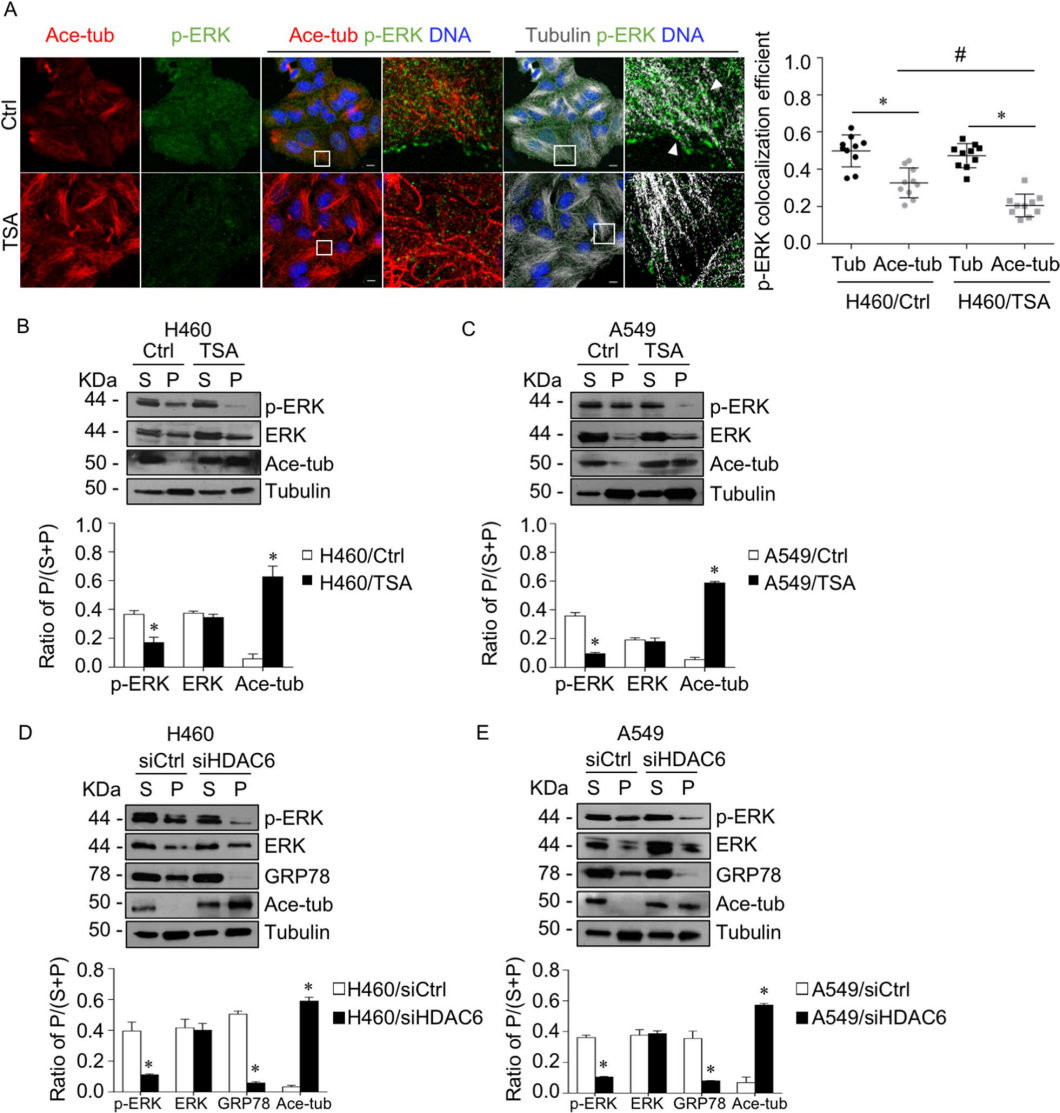
Suppression of HDAC6 prevented the microtubule-p-ERK interaction. **A** Immunofluorescence staining for p-ERK (green), acetylated tubulin (red), α-tubulin (gray), and DNA (blue) in H460 cells treated with or without TSA (5 µM TSA) for 4 h. Box areas are enlarged. The head arrows indicate p-ERK puncta overlapping with α-tubulin. Colocalization of p-ERK with acetylated tubulin or α-tubulin was calculated as Manders' coefficient. The plot shows individual data and is presented as the mean ± SEM. *p < 0.05; ^#^*p* < 0.05 (*n* = *10*). Scale bar is 10 µm. **B** H460 and **C** A549 cells were treated with 5 µM TSA for 4 h. The lysate was separated into soluble (S) and pellet (P) fractions using a microtubule sedimentation assay and analyzed for p-ERK, ERK, acetylated tubulin, and α-tubulin by immunoblotting. The ratio of the pellet to the total fraction was calculated. Data are presented as the mean ± SEM. *p < 0.05 vs. control cells (*n* = *3*). **D** H460 and **E** A549 cells were transfected with siRNA against HDAC6 (siHDAC6, 50 nM) or control siRNA (siCtrl). Cells were lysed, separated into soluble (S) and pellet (P) fractions using a microtubule sedimentation assay, and analyzed for p-ERK, ERK, acetylated tubulin, GRP78, and α-tubulin by immunoblotting. The ratio of the pellet to the total fraction was calculated. Data are presented as the mean ± SEM. *p < 0.05 vs. siCtrl cells (*n* = *3*)

HDAC6 is a unique functional enzyme with deacetylase activity against histones and other cytoplasmic substrates, including α-tubulin [9]. We tested whether HDAC6 inhibition induced tubulin acetylation, a major cytosolic substrate. Western blot analysis revealed that tubulin acetylation was significantly upregulated in a dose- (Fig. 3A, B) and time-dependent manner (Fig. 3C, D). Consistency, RNA interference to HDAC6 led to an increase in tubulin acetylation (Fig. 3E, F). Since accumulative studies demonstrated that microtubule post-translation modifications participated in cancer signaling, which they involved in the trafficking and stabilizing of molecules bound on them [39–41], we hypothesized that HDAC6 regulated ERK activity through tubulin acetylation, one of the microtubule post-translation modification types. First, an interaction of p-ERK with tubulins in the presence or absence of TSA was closely observed. Immunofluorescence revealed that p-ERK puncta were distributed throughout the cytoplasm and overlapped with α-tubulin, especially in the nonacetylated type (Fig. 4A). Its localization on tubulins was extensively reduced when tubulins were acetylated by TSA. The quantitative analysis showed a significant decrease in p-ERK and acetylated tubulin colocalization in TSA-treated cells compared to nontreated cells. To confirm this finding, p-ERK bound to microtubules was investigated by a microtubule sedimentation assay. The results demonstrated that the p-ERK accumulated in the microtubule fraction was significantly reduced in the TSA-treated group, which has high tubulin acetylation, whereas ERK was not altered (Fig. 4B, C). In addition, this phenomenon was also confirmed in HDAC6 knockdown cells that p-ERK was notably reduced in a microtubule compartment as compared to that of the control cells (Fig. 4D, E). This result suggested that microtubules were essential for p-ERK activity, in which microtubule acetylation prevented p-ERK localization and might facilitate p-ERK destabilization.

### GRP78 was required for p-ERK binding to microtubules

**Fig. 5:**
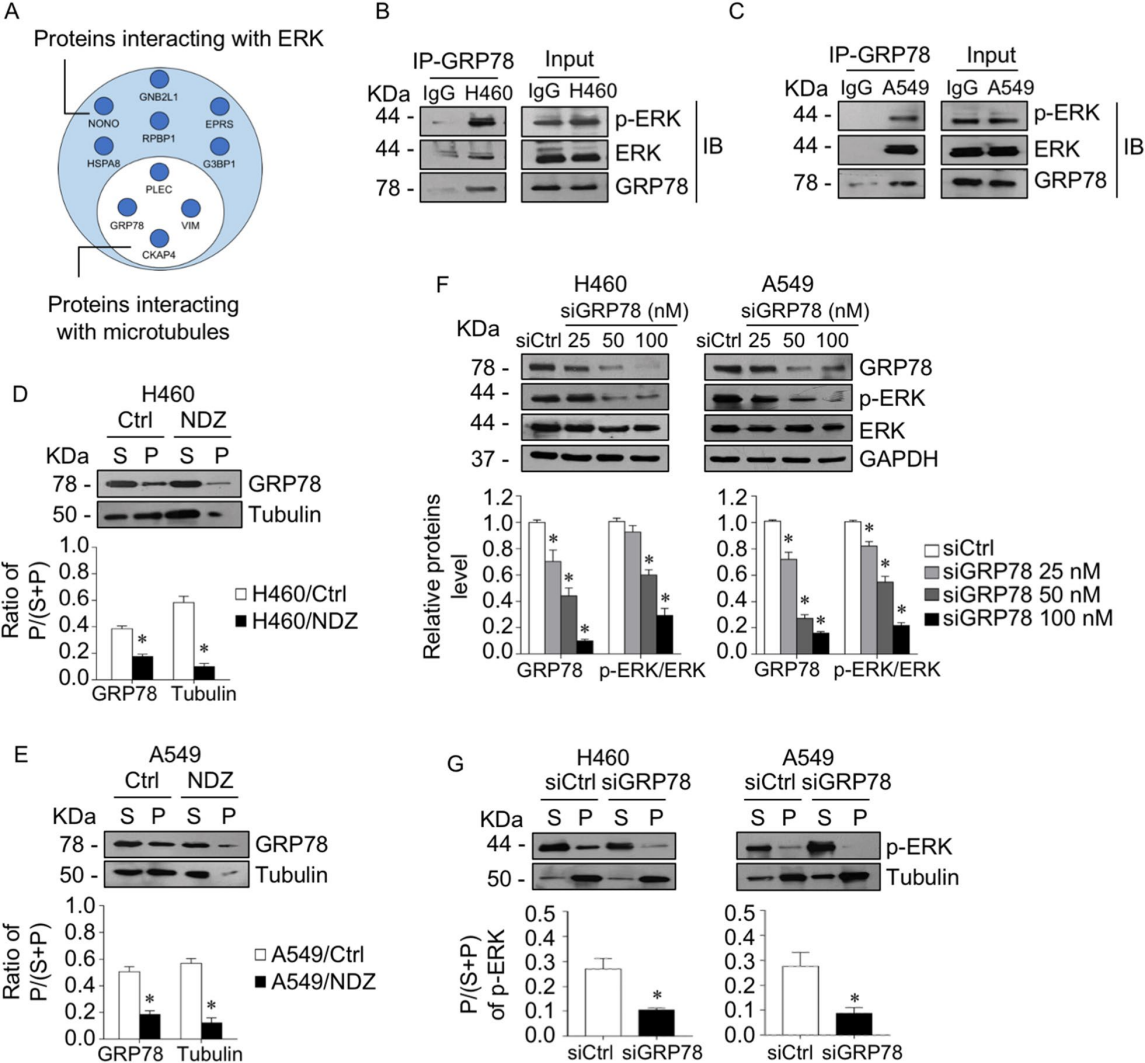
GRP78 was required for ERK phosphorylation stabilization on microtubules. **A** Circle diagram showing the peptides identified from immunoprecipitation and mass spectrometry (IP-MS) analysis. Proteins interacting with ERK were identified, in which microtubule-associated proteins are indicated (white circle). GRP78 was pulled down by a specific antibody or IgG as a negative control in **B** H460 and **C** A549 cells, and p-ERK, ERK, and GRP78 were evaluated by immunoblotting. Data were obtained from triplicate independent experiments. **D** H460 and **E** A549 cells were treated with or without 10 µM nocodazole (NDZ) for 1 h. Lysates were separated into soluble (S) and pellet (P) fractions using a microtubule sedimentation protocol and analyzed for GRP78 and α-tubulin by immunoblotting. The ratio of the pellet to the total fraction was calculated. Data are presented as the mean ± SEM. *p < 0.05 vs. control cells (*n* = *3*). **F** H460 and A549 cells were transfected with various concentrations of GRP78 siRNA (siGRP78) or control siRNA (siCtrl). The expression levels of GRP78, p-ERK, and ERK were analyzed by immunoblotting. The intensity was normalized to that of GAPDH. Data are presented as the mean ± SEM. *p < 0.05 vs. siCtrl cells (*n* = *3*). (**G**) GRP78 knockdown (siGRP78) and control (siCtrl) H460 and A549 cells were lysed, separated into soluble (S) and pellet (P) fractions using a microtubule sedimentation protocol, and analyzed for p-ERK and α-tubulin by immunoblotting. The ratio of the pellet to the total fraction was calculated. Data are presented as the mean ± SEM. *p < 0.05 vs. siCtrl cells (*n* = *3*)

To gain insight into the molecular mechanism by which HDAC6 regulates the p-ERK-microtubule interaction, we performed proteomic analysis to identify the potential microtubule-associated proteins that link p-ERK on microtubules. Immunoprecipitation was performed to pull down ERK and subjected to mass spectrometry analysis. The top 10 possible peptides interacting with ERK are shown, and the proteins that have been reported to be associated with microtubules were subclassified (Fig. 5A). The possible intermediate proteins interacting with both ERK and microtubules were consequently analyzed by the STRING database (Additional file 1: Table S5). The results demonstrated that the highest-ranking protein that possibly bound to ERK and microtubules was Plectin (PLEC), an intermediate filament-associated protein and signaling scaffolder [42]. Immunoprecipitation indicated that Plectin directly interacted with ERK and p-ERK (Additional file 1: Fig. S1A); however, p-ERK bound to microtubules was not affected by Plectin knockdown (Additional file 1: Fig. S1B, C).

Next, we tested another possible protein, glucose-regulated protein 78 (GRP78 or HSPA5), which was identified as an intermediate scaffold between ERK and microtubules (Fig. 5A, Additional file 1: Table S5). The results demonstrated that p-ERK coprecipitated with GRP78 (Fig. 5B, C). Furthermore, a microtubule sedimentation assay showed that GRP78 was present in both cytoplasmic and microtubule compartments, but after treatment with nocodazole (NDZ), a microtubule depolymerization agent, GRP78 in the microtubule fraction was clearly diminished, and the ratio of GRP78 in the pellet to the total fraction in both cell lines was extensively reduced (Fig. 5D, E). These data confirmed that GRP78 adhered to microtubules in lung adenocarcinoma cells. We further verified whether GRP78 functioned as a scaffold for p-ERK localized on microtubules. GRP78 was knocked down in H460 and A549 cells, and the results demonstrated that p-ERK levels were gradually downregulated in accordance with GRP78 levels (Fig. 5F). Interestingly, the microtubule sedimentation assay revealed that the ratio of p-ERK in the microtubules to the total fraction was strongly decreased in GRP78 knockdown cells compared to mock control cells (Fig. 5G). Taken together, these results indicated that GRP78 was required for p-ERK binding to microtubules in lung cancer cells.

### Suppression of HDAC6 by TSA and siHDAC6 caused p-ERK-GRP78 to detach from microtubules

**Fig. 6:**
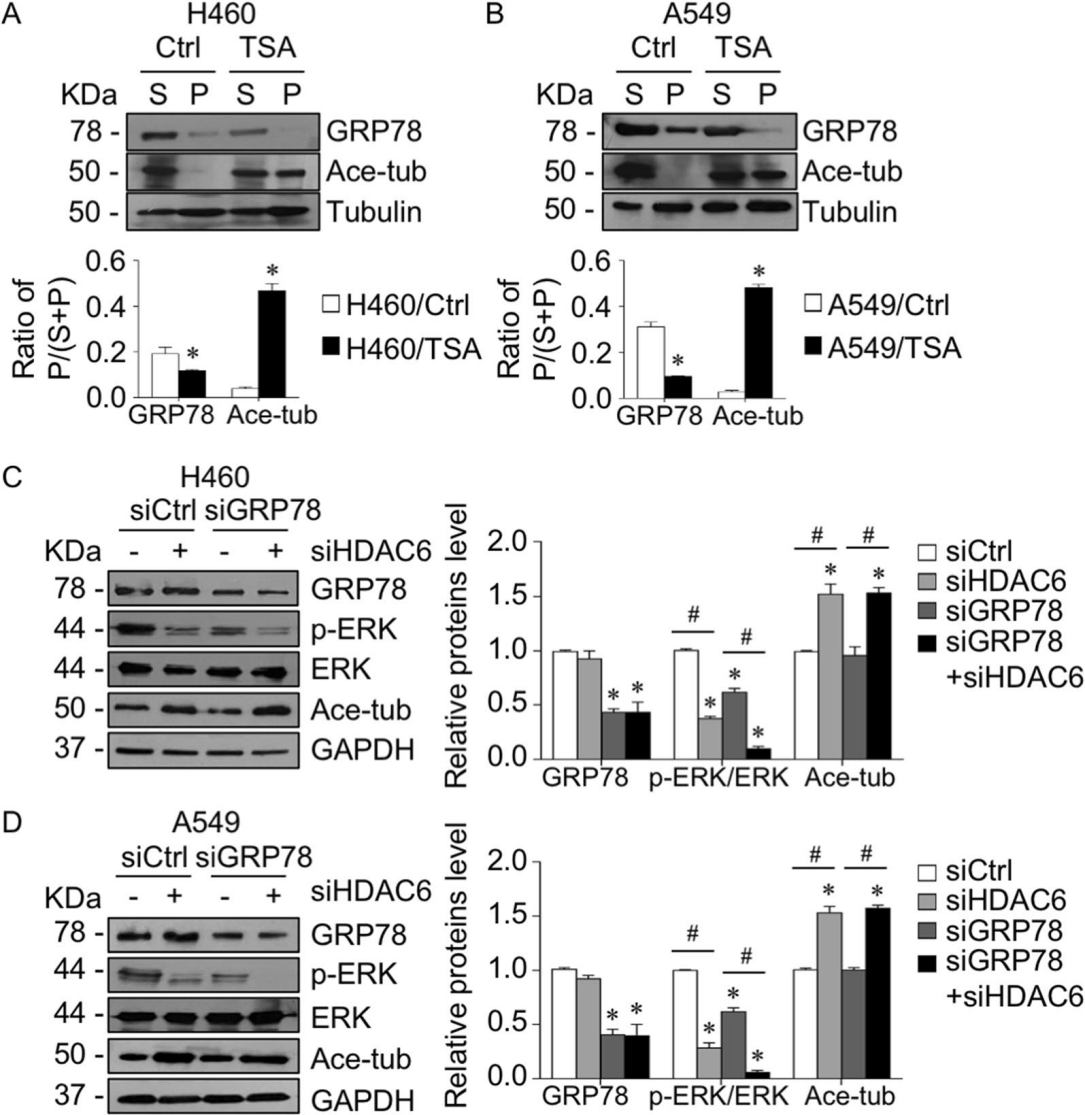
Suppression of HDAC6 mediated GRP78-p-ERK detachment from microtubules. **A** H460 and **B** A549 cells were treated with 5 µM TSA for 4 h. Lysates were separated into soluble (S) and pellet (P) fractions using a microtubule sedimentation assay and analyzed for GRP78, acetylated tubulin, and α-tubulin by immunoblotting. The ratio of the pellet to the total fraction was calculated. Data are presented as the mean ± SEM. *p < 0.05 vs. control cells (*n* = *3*). **C** H460 and **D** A549 cells were transfected with siHDAC6, siGRP78, or control siRNA (siCtrl). The expression levels of GRP78, p-ERK, ERK, and acetylated tubulin were analyzed by immunoblotting. The intensity was normalized to that of GAPDH. Data are presented as the mean ± SEM. *p < 0.05 vs. siCtrl cells; ^#^*p* < 0.05 vs indicated cells (*n* = *3*)

Our findings showed that either suppression of HDAC6 activity by TSA or its expression by siHDAC6 inhibited ERK phosphorylation by upregulating tubulin acetylation and that GRP78 was required for the microtubule-ERK interaction. We hypothesized that the suppression of HDAC6 by TSA and siHDAC6 prevented GRP78 localization on microtubule acetylation, consequently mediating p-ERK detachment from microtubules and destabilization. A microtubule sedimentation assay was conducted in the presence or absence of TSA. In the microtubule compartment, GRP78 accumulation was notably decreased in the TSA-treated cells, which have high tubulin acetylation, compared to the control, suggesting that GRP78 was not localized preferentially on microtubule acetylation (Fig. 6A, B). Similarly, HDAC6 knockdown caused a decline in GRP78 level in the microtubule compartment as compared to the control cells (Fig. 4D, E). Immunofluorescence also showed that TSA treatment caused a detachment of p-ERK-GRP78 complexes from microtubules (Additional file 1: Fig. S2). In addition, silencing GRP78 caused a reduction in p-ERK compared to control siRNA, and this effect was markedly potentiated in the combination with siHDAC6 (Fig. 6C, D). Taken together, these results indicated that HDAC6 might exert p-ERK activity by stabilizing p-ERK on nonacetylated microtubules, in which GRP78 was necessary for its localization on microtubules. HDAC6 suppression resulted in an increase in acetylated tubulin and p-ERK-GRP78 dissociation from microtubules and consequent ERK deactivation.

### Downregulation of HDAC6 suppressed lung cancer growth through a GRP78-ERK-dependent mechanism

**Fig. 7:**
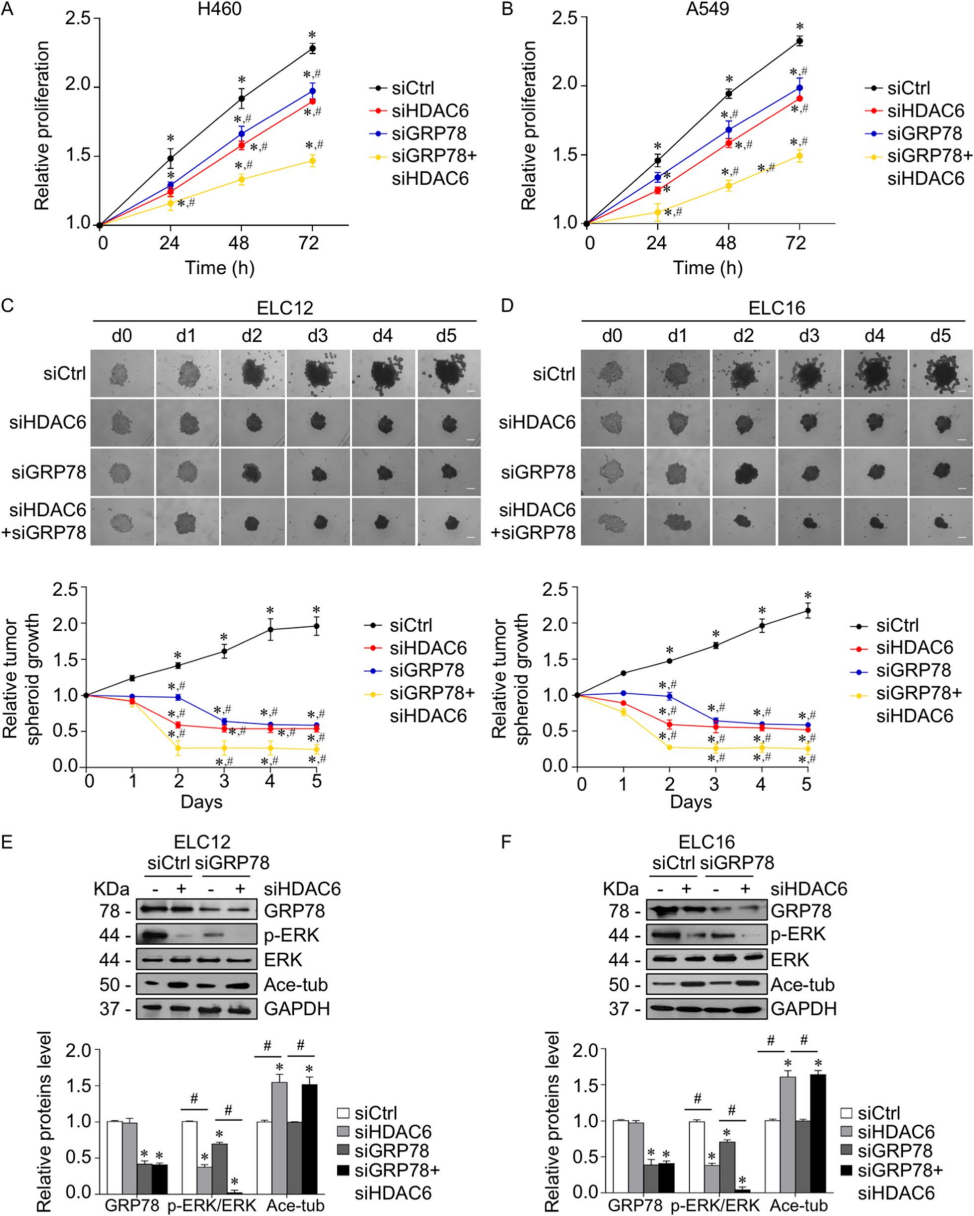
HDAC6 silencing attenuated lung cancer growth through a GRP78-ERK-dependent mechanism. **A** H460 and **B** A549 cells were transfected with siHAC6, siGRP78, or control siRNA (siCtrl). Cell proliferation was examined by an MTT assay. Data are presented as the mean ± SEM. *p < 0.05 vs. initial time point (0 h); ^#^*p* < 0.05 vs. siCtrl group (*n* = *3*). Patient-derived lung cancer **C** ELC12 and **D** ELC16 cells were transfected siHDAC6, siGRP78, or control siRNA (siCtrl), and seeded onto ultralow attachment plates for allowing 3D tumor spheroid formation. The tumor spheroid growth rate was quantified from the spheroid diameter using ImageJ software and calculated relative to that of the initial time point. Data are presented as the mean ± SEM. *p < 0.05 vs. initial time point (0 h); ^#^*p* < 0.05 vs. siCtrl group (*n* = *3*). Scale bar is 100 µm. After 5 d of inoculation, tumor spheroids growth from transfectant **C** ELC12 and **D** ELC16 cells were lysed. The expression levels of GRP78, p-ERK, ERK, and acetylated tubulin were analyzed by immunoblotting. The intensity was normalized to that of GAPDH. Data are presented as the mean ± SEM. *p < 0.05 vs. siCtrl cells; ^#^*p* < 0.05 vs. between indicated groups (*n* = *3*)

Since HDAC6 plays an important role in lung cancer pathogenesis, we further investigated whether suppression of HDAC6 by siHDAC6 impeded lung cancer growth via a GRP78-dependent mechanism. An in vitro 2D cell proliferation assay demonstrated that HDAC6 knockdown caused cell growth retardation compared to the control group, and the effect of siHDAC6 was notably potentiated in GRP78 knockdown cells (Fig. 7A, B). Moreover, it is widely accepted that 3D tumor spheroid formation mimics the pathophysiology of in vivo tumor growth and is a reliable technique for the preclinical evaluation of cancer growth activity [43]. We further investigated the role of HDAC6 in 3D tumor spheroid growth in patient-derived primary lung cancer. The 3D spheroid formation was established using an ultralow attachment plate, and their initial growth was monitored. After 5 d of inoculation, either HDAC6 or GRP78 silencing showed a gradual reduction in the tumor spheroid growth rate, while double knockdown of both HDAC6 and GRP78 exhibited the greatest inhibitory effect (Fig. 7C, D), corresponding to an increase in tubulin acetylation and a decline in p-ERK expression (Fig. 7E, F). Taken together, our results suggested that suppression of HDAC6 attenuated lung cancer growth via a GRP78-ERK-dependent mechanism.

## Discussion

The present study revealed the biological role of HDAC6 in lung cancer cell growth and its molecular mechanism. HDAC6 inhibition caused tubulin hyperacetylation, consequently facilitating p-ERK detachment from microtubules, in which GRP78 was required for p-ERK stabilization on nonacetylated tubulin. HDACs are key enzymes of the transcriptional cofactors controlling gene expression via deacetylation of lysine residues on their substrates [13]. HDACs are also widely associated with cell differentiation and proliferation through the regulation of gene transcription [6, 13]. Accumulating studies have shown that HDAC6 is overexpressed in numerous cancers and correlates with cancer prognosis [14–16]. Consistent with our study, HDAC6 was significantly elevated in lung cancer cells and malignant tissues, and HDAC6 inhibition was able to attenuate lung cancer cell growth, suggesting HDAC6 as a potential prognostic/therapeutic target in lung cancer.

HDAC overexpression was shown to promote cancer cell growth by repressing growth-suppressive genes [44]. Previous studies reported that the recruitment of HDACs to the target promotor can inhibit the transcription of specific cancer genes, including proapoptotic Bax, cyclin-dependent kinase inhibitor p21, and receptors for the growth-restraining signaling molecule TGF-βRII [45, 46], contributing to the promotion of cancer cell growth. Among the subclasses, class IIB HDACs, including HDAC6, are located in the cytoplasm and function to maintain the balance of nonhistone protein acetylation, such as α-tubulin [6]. Under high acetylated conditions, microtubules become more stable; in contrast to dynamic microtubules, they are predominantly hypoacetylated. Interference with HDAC6 activity and/or expression affects microtubule acetylation and subsequently several biological processes [12, 13, 17]. Similar to our findings, HDAC6 silencing enhanced tubulin acetylation and thus attenuated lung cancer growth, indicating that HDAC6 functions as a tumor promotor.

ERK plays an important role in cancer signaling that regulates cancer proliferation, growth, and survival [21, 22]. The active status of ERK is mainly dominated by the balance between its phosphorylation and dephosphorylation, in which the dephosphorylation process is regulated by the alkaline phosphatase enzyme, while phosphorylation is mediated through an upstream cascade kinase [47]. After ERK is activated by mitogenic stress or conditional active Raf-1 and MEK, phosphorylated ERK translocates to the nucleus and promotes the transcription of genes associated with cancer progression [21]. Although canonical ERK signal transduction is governed by the phosphorylation process, its post-translational modifications (PTMs) are important in ERK activity [21]. The elements of the ERK cascade can be manipulated by HDAC6 in numerous biological processes [24, 27, 28]. However, the connection between its deacetylase activity and the kinase function of ERK remains largely unknown. Based on previous studies, we hypothesized that HDAC6 regulated ERK activation at least in part through an HDAC6 substrate due to its absence of kinase enzymatic function. Among HDAC6 substrates, α-tubulin is widely known to be involved in the modulation of several cancer signaling pathways via its PTM process, including acetylation [39, 41, 48]. A recent study showed that acetylated tubulin mediated EMT in lung cancer cells through the CAMSAP3/Akt axis [39]. Tubulin acetylation promoted and preserved active Akt, an EMT-promoting factor; in contrast, deacetylation of tubulin caused p-Akt inactivation. As cellular trafficking components, microtubules regulate the translocation and stabilization of their cargoes, such as organelles and signaling molecules, to the specific targeted site through their dynamic properties, which are controlled by microtubule PTMs [39, 40, 49]. A high level of α-tubulin acetylation mediates kinesin-1 and JIP-1 binding to microtubules, facilitating JNK phosphorylation and activation, which are necessary for autophagosome formation in cervical cancer [48]. In contrast, under low shear stress, as occurs during systemic circulation, tubulin acetylation is reduced, which induced the endocytic trafficking of integrin β1, thereby enhancing focal adhesion turnover and breast cancer cell migration [41], suggesting that cellular trafficking regulated by tubulin acetylation is dependent on specific cell conditions, cargoes and microtubule-associated proteins. In addition, it has been reported that microtubules stabilize p-ERK bound to them by preventing it from dephosphorylation [50, 51]. Concurrent with our study, microtubules, particularly the nonacetylated type, were required for the regulation of HDAC6 on ERK activation. When the HDAC6 status was normal, p-ERK bound to and was stabilized on microtubules, and after the microtubules became more acetylated as mediated by HDAC6 suppression, p-ERK detached and was destabilized.

Interestingly, proteomic analysis revealed GRP78 as an intermediate scaffold regulating p-ERK localization on microtubules. GRP78 is an endoplasmic reticulum (ER)-resident molecular chaperone involved in correcting misfolded proteins in the ER [52]. In addition to maintaining protein stability, cytoplasmic and cell surface GRP78 participate in several signaling pathways [53–55], and its high expression has been reported in many cancer types facilitating tumor progression [56, 57]. Cell surface GRP78 stimulated cell migration and invasion by directly interacting with the extracellular matrix (ECM) adhesion molecule β1-integrin and facilitating ECM degradation [53]. GRP78 promoted invasion by inducing the secretion of MMPs in a FAK/JNK-dependent mechanism [54] and correlated with poor prognosis in pancreatic cancer [56]. A recent study reported that GRP78 significantly enhanced ERK signaling and consequently promoted the EMT process [58]; however, the underlying mechanism by which GRP78 regulates ERK has not yet been elucidated. In linkage with HDAC6, HDAC6 inhibition was shown to mediate GRP78 acetylation and consequently suppress GRP78 translocation to the cell surface [55], suggesting that microtubules might be implicated in this process. Their clinical association also revealed that patients with high HDAC6 and GRP78 levels had the lowest overall survival rate than the other groups (Additional file 1: Fig. S4). Additionally, our present study demonstrated that suppression of HDAC6 upregulated acetylated tubulin, and subsequently, p-ERK and GRP78 were dissociated, leading to a decrease in ERK activity.

It is possible that acetylated GRP78 mediated by HDAC6 inhibition might interfere with its scaffold function for p-ERK associating on microtubules since it has been reported that HDAC6 inhibitor mediated GRP78 acetylation and affected the GRP78 interaction with its partner [59]. Our data also showed that HDAC6 knockdown caused GRP78 acetylation (Additional file 1: Fig. S3). However, GRP78 knockdown alone has less efficacy on p-ERK reduction and tumor growth than those of the siHDAC6 group, and the combination with siHDAC6 remarkedly potentiated this effect (Figs. 6C, D, 7). It suggested that GRP78 acetylation mediated by siHDAC6 might partially participate in its scaffold function for p-ERK localizing on microtubules, and tubulin acetylation caused by HDAC6 suppression plays a major regulator in this phenomenon.

Several studies had reported an in vivo anti-cancer activity of HDAC6 inhibitor. Previous study demonstrated that treatment with TSA, a HDAC6 inhibitor, strongly suppressed the growth of lung tumor xenograft significantly [60]. TSA also effectively suppressed A549 tumor xenograft, which exerted the anti-cancer effect Bovine herpesvirus 1 (BoHV-1) infection [61]. We further extend the investigation using patient-derived cancer cells in combination with an in vitro 3D tumorigenesis model. In vitro 3D-tumorigenesis model is a promising platform that mimics both the structural architecture of malignancy and characteristics of cancer microenvironment such as cell–cell interaction and the secretion of cytokines and provides a powerful tool for an assessment of new drugs or compounds at the pre-clinical stage of drug discovery [62–64]. In addition, the experiment in patient-derived cancer cells is an alternative approach for evaluation of anti-cancer therapeutics, which this model exhibits an actual genetic information of human, providing precise molecular mechanism and accurate clinical response [29, 65]. Our results demonstrated that siHDAC6 strongly suppressed in vitro 3D-tumorigenesis, which was exerted in the present of siGRP78 (Fig. 7), supporting the biological role of HDAC6 and its potential therapeutic target. This study first reported that cytoplasmic GRP78 regulates ERK activity in a microtubule-dependent manner, which reveals an oncogenic role of HDAC6, filling the research gap in cancer cell biology. Further in-depth for investigation of HDAC6-GRP78 activity using an in vivo model and the investigation of how GRP78 preserves ERK activation, whether it is involved in preventing dephosphatase activity, is necessary to support its potential clinical application.

## Conclusion

**Fig. 8:**
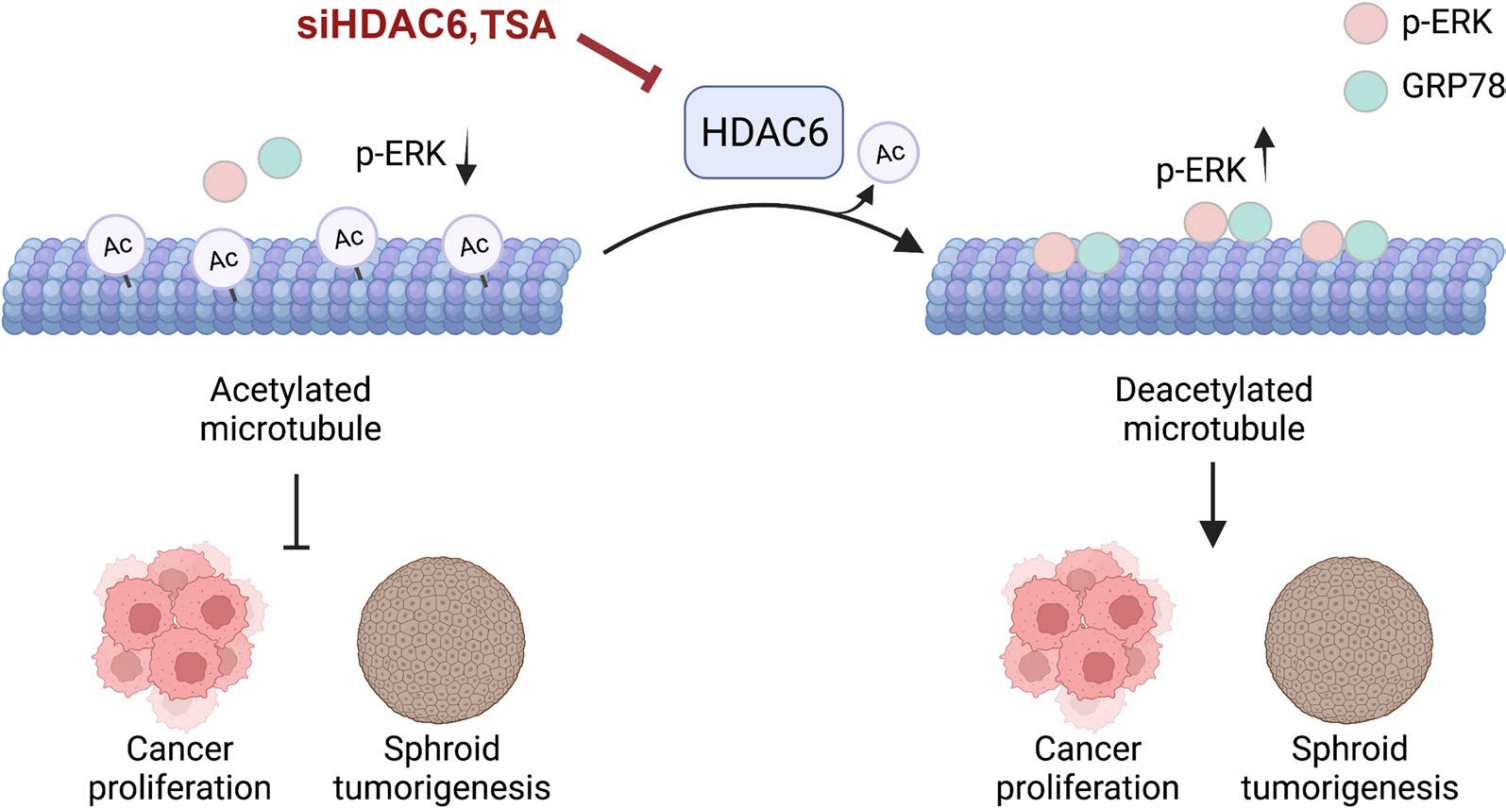
Schematic model of HDAC6 regulating lung cancer cell growth. HDAC6 functions to deacetylate tubulin, on which the p-ERK/GRP78 complex preferentially localizes, which is required for stabilizing ERK activity. Suppression of HDAC6 by TSA and siHDAC6 caused hyperacetylated tubulin and consequently p-ERK/GRP78 dissociation from microtubules. ERK activity is destabilized and decreased, leading to suppression of cancer cell proliferation and spheroid formation

The poor prognosis of lung cancer patients remains an indispensable clinical concern. A better understanding of the molecular mechanism provides a therapeutic approach to improve clinical outcomes. Herein, we reported that HDAC6 was upregulated in lung cancer and strongly correlated with poor prognosis. Mechanistically, HDAC6 inhibition was able to attenuate lung cancer growth by upregulating tubulin acetylation and downregulating p-ERK (Fig. 8). p-ERK localized on microtubules, and GRP78 was an intermediate scaffold required for this process. Upon microtubule hyperacetylation, the GRP78/p-ERK complex dissociated from microtubules, leading to p-ERK destabilization and thereby inhibiting in vitro 2D and 3D lung cancer growth. These findings suggest that HDAC6 is a potential therapeutic target, and either suppressing its function or expression provides a promising therapeutic strategy against lung cancer.

## Supplementary Information

**Additional file 1. **Supplementary Method: RNA interference (RNAi) to Plectin and transfection. **Table S1.** List of the primer sequences used for RT–PCR analysis in this study. **Table S2.** List of the primary and secondary antibodies, their company, catalog number, host species, and working dilution. **Table S3.** The clinical characteristics of lung cancer patients. **Table S4.** The combined score of the cancer signaling-related proteins of HDAC6 obtained from the STRING database. **Table S5.** The combined score of the ERK-microtubule interacting peptides from STRING database. **Fig. S1.** ERK phosphorylation localized on microtubules was not regulated by Plectin. **Fig. S2.** HDAC6 inhibition prevented the p-ERK-GRP78 complexes localized on microtubules. **Fig. S3.** HDAC6 RNA interference mediated GRP78 acetylation. **Fig. S4.** High HDAC6 and GRP78 expressions were significantly associated with poor prognosis.

## Data Availability

All data generated or analyzed during this study are included in this published article and supplementary information file.

## Abbreviations

EMT: Epithelial to mesenchymal transition
ERK: Extracellular signal-regulated kinase
GRP78: Glucose-regulated protein 78
HDAC6: Histone deacetylase 6
PTMs: Post-translational modifications
TSA: Trichostatin A
