# Supplementary material for: Inhibition of histone deacetylase 6 destabilizes ERK phosphorylation and suppresses cancer proliferation via modulation of the tubulin acetylation-GRP78 interaction
Source: J Biomed Sci. 2023 Jan 13;30:4. doi: 10.1186/s12929-023-00898-3 (PMC9838051; doi:10.1186/s12929-023-00898-3)
Supplement: Supplementary file 1 — Additional file 1. Supplementary Method: RNA interference (RNAi) to Plectin and transfection. Table S1. List of the primer sequences used for RT–PCR analysis in this study. Table S2. List of the primary and secondary antibodies, their company, catalog number, host species, and working dilution. Table S3. The clinical characteristics of lung cancer patients. Table S4. The combined score of the cancer signaling-related proteins of HDAC6 obtained from the STRING database. Table S5. The combined score of the ERK-microtubule interacting peptides from STRING database. Fig. S1. ERK phosphorylation localized on microtubules was not regulated by Plectin. Fig. S2. HDAC6 inhibition prevented the p-ERK-GRP78 complexes localized on microtubules. Fig. S3. HDAC6 RNA interference mediated GRP78 acetylation. Fig. S4. High HDAC6 and GRP78 expressions were significantly associated with poor prognosis. [file 12929_2023_898_MOESM1_ESM.docx]

***Additional file for***

**Inhibition of histone deacetylase 6 destabilizes ERK phosphorylation and suppresses cancer proliferation via modulation of the tubulin acetylation-GRP78 interaction**

Onsurang Wattanathamsan ^1^, Naphat Chantaravisoot^2,3^, Piriya Wongkongkathep^3^, Sakkarin Kungsukool^4^, Paninee Chetprayoon^5^, Pithi Chanvorachote^1^, Chanida Vinayanuwattikun^6^, Varisa Pongrakhananon^1,7*^

^1^ Department of Pharmacology and Physiology, Faculty of Pharmaceutical Sciences, Chulalongkorn University, Bangkok, Thailand

^2^ Department of Biochemistry, Faculty of Medicine, Chulalongkorn University, Bangkok, Thailand

^3^ Center of Excellence in Systems Biology, Faculty of Medicine, Chulalongkorn University, Bangkok, Thailand

^4^ Department of Respiratory Medicine, Central Chest Institute of Thailand, Muang District, Nonthaburi, Thailand

^5^ Toxicology and Bio Evaluation Service Center, National Science and Technology Development Agency, Pathum Thani, Thailand

^6^ Division of Medical Oncology, Department of Medicine, Faculty of Medicine, Chulalongkorn University, Bangkok, Thailand

^7^ Preclinical Toxicity and Efficacy Assessment of Medicines and Chemicals Research Cluster, Chulalongkorn University, Bangkok, Thailand

Correspondence: Varisa Pongrakhananon, Department of Pharmacology and Physiology, Faculty of Pharmaceutical Sciences, Chulalongkorn University, Bangkok, Thailand

Email: varisa.p@pharm.chula.ac.th

**Supplementary method**

**RNA interference (RNAi) to Plectin and transfection**

The knockdown experiment was performed using Lipofectamine® RNAiMAX following the manufacturer’s instruction (Invitrogen, MA, USA). Stealth RNAi and control were purchased from Invitrogen (Invitrogen, MA, USA) which the sequence of siPlectin was 5’- CAAGGTGTACCGGCAGACCAACCTG-3’ Briefly, 25 nM of siRNA in OptiMEM (Invitrogen, MA, USA) were incubated with Lipofectamine® RNAiMAX in OptiMEM at room temperature for 15 min. The mixture was dropped wisely onto the cells and incubated further at 37 °C for 6 h. After transfection for 72 h, transfection efficiency was assessed by western blot analysis, prior to other biochemical assays.

**Table S1** List of the primer sequences used for RT–PCR analysis in this study

| **Genes** | **Primer sequences (5'-3')** | |
| --- | --- | --- |
|  | **F’: Forward primer** | **R’: reverse primer** |
| HDAC6 | F’: AAGAAGACCTAATCGTGGGACT | R’: GCTGTGAACCAACATCAGCTC |
| GAPDH | F’: ACATCGCTCAGACACCATG | R’: TGTAGTTGAGGTCAATGAAGGG |

**Table S2** List of the primary and secondary antibodies, their company, catalog number, host species, and working dilution.

| **Antibody** | **Company** | **Catalog number** | **Host species** | **Working Concentration** | |
| --- | --- | --- | --- | --- | --- |
|  |  |  |  | **WB** | **IF** |
| HDAC6 | Cell Signaling Technology | #7558 | Rabbit | 1:2000 | - |
| p-ERK | Cell Signaling Technology | #4376 | Rabbit | 1:1000 | - |
| p-ERK | Cell Signaling Technology | #9101 | Rabbit | - | 1:1000 |
| ERK | Cell Signaling Technology | #4695 | Rabbit | 1:1000 | - |
| Acetylated tubulin | Sigma | #T7451 | Mouse | 1:5000 | 1:1000 |
| Acetylated lysine | Cell Signaling Technology | #9441 | Rabbit | 1:1000 | - |
| GRP78 | Santa Cruz Biotechnology | #sc-166490 | Mouse | 1:1000 | - |
| Plectin | Abcam | #AB-AB32528 | Rabbit | 1:2000 | - |
| Tubulin | Sigma | #T6199 | Mouse | 1:5000 | - |
| GAPDH | Cell Signaling Technology | #97166 | Mouse | 1:1000 | - |
| α-tubulin | EMD Millipore | #MAB1864 | Rat | - | 1:1000 |
| Alexa Fluor 488 | Invitrogen | #A11034 | Rabbit | - | 1:1000 |
| Alexa Fluor 568 | Invitrogen | #A11032 | Mouse | - | 1:1000 |
| Alexa Fluor 647 | Invitrogen | #A21247 | Rat | - | 1:1000 |
| HRP-conjugated anti-rabbit | Cell Signaling Technology | #7074 | Rabbit | 1:1000 | - |
| HRP-conjugated anti-Mouse | Cell Signaling Technology | #7076 | Mouse | 1:1000 | - |

**Table S3** Clinical characteristics of lung cancer patients.

| **Characteristics** | **Malignancy  (n = 22)** | **Benign  (n=21)** | ***P* value*** |
| --- | --- | --- | --- |
| **Sex ― no. (%)** | | | |
| Female | 8 (36.36) | 11 (52.38) | 0.2904 |
| Male | 14 (63.64) | 10 (47.62) |  |
| **Age ― yr** | | | |
| Mean (SD) | 64.84 (11.45) | 50.94 (15.41) | 0.6141 |
| Median (Min, Max) | 67 (42, 87) | 57 (17, 74) |  |
| **Tobacco history — no. (%)** | | | |
| Smoker | 12 (54.54) | 6 (28.57) | 0.0844 |
| Non-smoker | 10 (45.45) | 15 (71.43) |  |
| **Stage — no. (%)** | | | |
| I  IIA  IIB  IIIA  IIIB  IIII | 3 (13.64)  3 (13.64)  3 (13.64)  5 (22.73)  3 (13.64)  5 (22.73) |  |  |

* Calculated based on Mann-Whitney U test and Chi-square test.

**Table S4** The combined score of the cancer signaling-related proteins of HDAC6 obtained from the STRING database

| **Node1** | **Node 2** | **Combined score** |
| --- | --- | --- |
| HDAC6 | AKT1 | 0.704 |
| HDAC6 | MAPK1 | 0.584 |
| HDAC6 | TNF | 0.403 |

**Table S5** The combined score of the ERK-microtubule interacting peptides from STRING database

| **Node1** | **Node 2** | **Combined score** |
| --- | --- | --- |
| TUBB | PLEC | 0.552 |
| TUBB | HSPA5 | 0.438 |
| TUBB | VIM | 0.355 |
| MAPK1 | PLEC | 0.354 |
| MAPK1 | HSPA5 | 0.295 |
| MAPK1 | VIM | 0.283 |
| MAPK1 | TUBB | 0.267 |

**
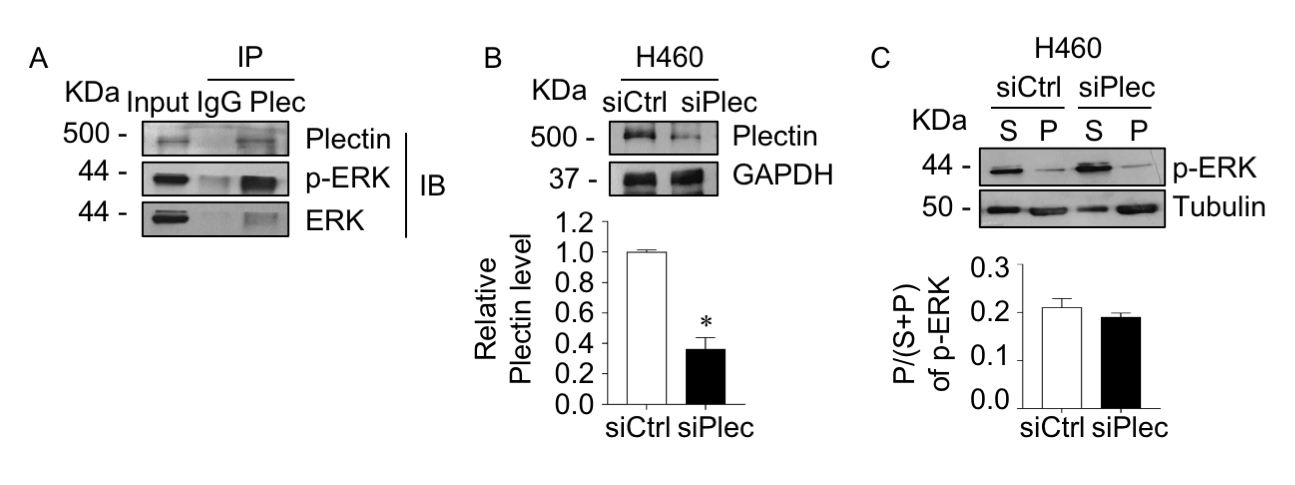
**

**Fig. S1** ERK phosphorylation localized on microtubules was not regulated by Plectin. (**A**) H460 cells were pulled down with an antibody against Plectin or IgG as a negative control and then immunoblotted for p-ERK, ERK, and Plectin. Data were obtained from triplicate independent experiments. (**B**) H460 cells were transfected with siRNA against Plectin (siPlec) or control siRNA (siCtrl). Transfection efficiency was analyzed by immunoblotting. The intensity was normalized to that of GAPDH. Data are presented as the mean ± SEM. *, *p* < 0.05 vs. siCtrl cells (*n=3*). (**C**) Plectin knockdown (siPlec) and control (siCtrl) H460 cells were lysed, separated into soluble (S) and pellet (P) fractions using a microtubule sedimentation protocol and analyzed for p-ERK and α-tubulin by immunoblotting. The ratio of the pellet to the total fraction was calculated. Data are presented as the mean ± SEM. *, *p* < 0.05 vs. siCtrl cells (*n=3*).

**
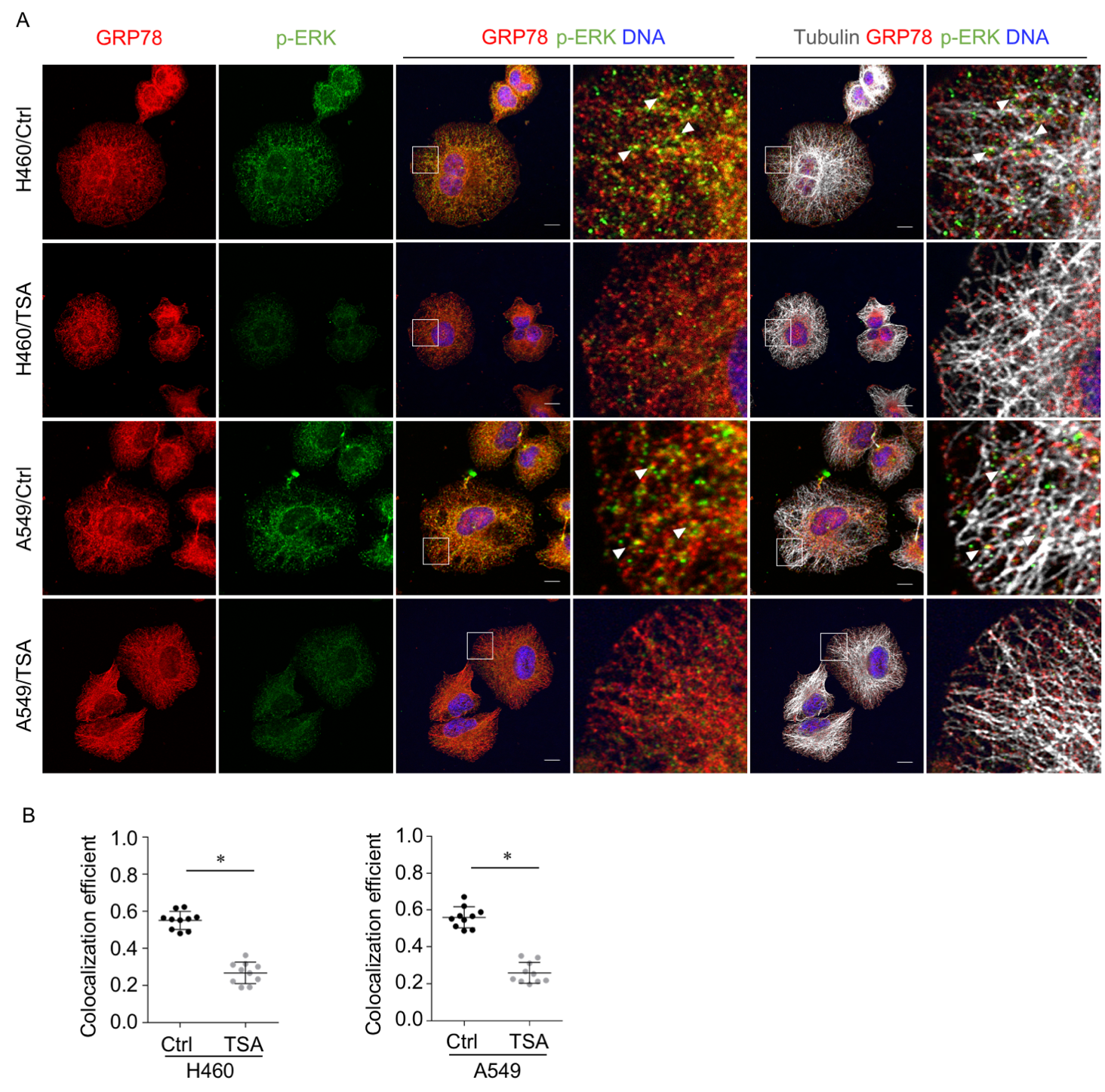
**

**Fig. S2** HDAC6 inhibition prevented the p-ERK-GRP78 complexes localized on microtubules. (**A**) Immunofluorescence staining for p-ERK (green), GRP78 (red), α-tubulin (gray), and DNA (blue) in H460 and A549 cells treated with or without TSA (5 µM TSA) for 4 h. Box areas are enlarged. The arrows indicate p-ERK puncta overlapping with GRP78 puncta. (**B**) Colocalization of p-ERK-GRP78 complexes on microtubules was calculated as Manders' coefficient. The plot shows individual data and is presented as the mean ± SEM. *, *p* < 0.05 vs. Ctrl cells (*n=10*). Scale bar is 10 µm.


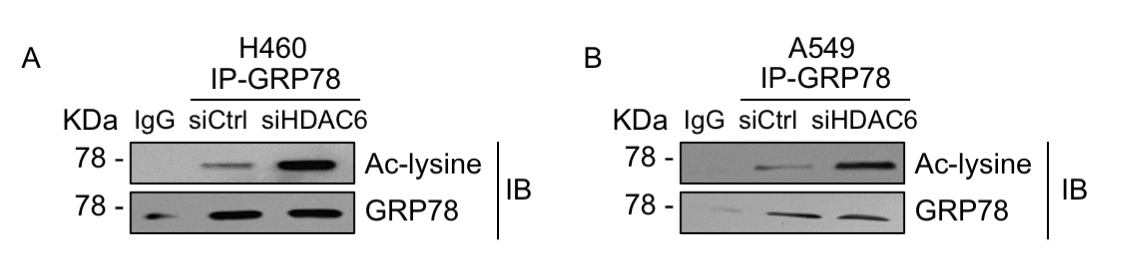


**Fig. S3** HDAC6 RNA interference mediated GRP78 acetylation. H460 (A) and A549 (B) cells were transfected with either siRNA to HDAC6 or control. Cell lysates were pulled down with anti-GRP78 and immunoblotted with anti-acetylated lysine (Ac-lysine) and anti-GRP78. The represented blot from three independent experiments was shown.

**
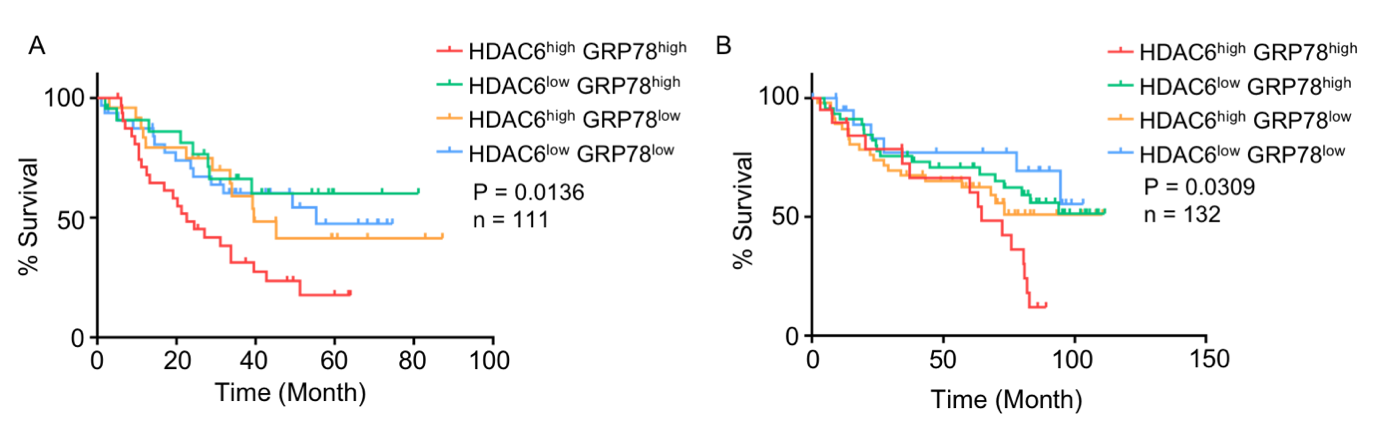
**

**Fig. S4** High HDAC6 and GRP78 expressions were significantly associated with poor prognosis. Kaplan–Meier survival analysis according to HDAC6 and GRP78 levels in a lung adenocarcinoma obtained from GEO dataset (A) GSE3141 and (B) GSE14814 was performed. Lung cancer patients with high HDAC6/high GRP78 (red) was associated with the worse overall survival among other groups. Survival curves of patients whose tumors exhibited low HDAC6/high GRP78 expressions (green), high HDAC6/low GRP78 expression (yellow) and low HDAC6/low GRP78 expression (blue) are indicated.
